# Mr. Wawn, on Filling and Extracting Teeth

**Published:** 1807-09

**Authors:** Charles N. Wawn

**Affiliations:** Newcastle, Tyne


### *To the Editors of the Medical and Physical Journal.*

Gentlemen,

In the second Volume of Fox's Work on the Teeth, recently published, mention is made of *a new method of stopping teeth* by means of "the fusible," (or Newton's) "metal," which had been suggested to Mr. F. by "some chemical

---

doubtedly very considerable, but much too indefinitive to admit ot being estimated with any degree of practical precision. The testimony, however, here adduced, brings into question the justness of T. Y's report, that fewer inconveniences arose from the use of *turpentine* than from that of *cold water*. Dr. K.

mical gentlemen," (in another place attributed to Mr. Pepys,) and which he thinks "promises to be very successful" in certain cases. Now it happened that the same idea occurred to *me* nearly three years ago, though I confess with much more moderate expectations of its success; but as I have since been induced to abandon all hopes of its practical utility, I trust I may be allowed to state my reasons.

In several teeth which I filled up in this manner, *(ex capite)* the degree of heat, though very small, compared with that requisite for the fusion of metals in general, was yet so considerable to my feelings, and so permanent, as to render it almost impossible for me, in filling a large cavity, to retain the tooth between my fingers until it became cool; and of consequence, I never considered myself warranted in making an experiment of the kind on a patient, when the probable result would have been such a degree of pain and inflammation, as would have completely frustrated my purpose. Another objection which I conceive to be fatal to it is, that in common with all other metals, in passing from the state of fusion to that of congelation, it contracts in bulk, and consequently cannot, when poured into a tooth, prove that "most perfect mode of filling up" that Mr. F. supposes. There are also several *minor* difficulties attending it, as the morbid sensibility that is frequently present in carious teeth; the decayed cavity opening on the side of a tooth, or between two teeth, &c.; and in the bulk of the most favourable cases in the *upper teeth*, it would be wholly inadmissible without the awkward circumstance of *inverting* the patient.

I will avail myself of this opportunity of mentioning an addition to the key instrument which I have been for some time in the habit of using; and though a very simple one, I have found it highly useful on many occasions. It consists merely in having *an ordinary claw made with a spreading point*, so as to be about one-third of an inch broad, (or the breadth of a common molaris) at its termination. By it, the *utmost possible hold* is obtained of the fangs of a tooth, when the crown is perhaps almost wholly gone, and their removal *at once* effected; thus, saving the patient, the pain and the operator the discredit of a second application of the instrument, where the ordinary narrow-pointed claw could at most have removed but *one* of the fangs at once, and that perhaps at the risk of not getting a firm hold of either. The discretion of the Practitioner must guide him as to the cases in which it is applicable; but in the course of between two and three years frequent use usage of it, I have on many occasions experienced in it a most decided advantage over the common claw

CHARLES N. WAWN.

*Newcastle, Tyne, Aug.* 10, 1807.

